# Morphological and biomolecular targets in retina and vitreous from Reelin-deficient mice (Reeler): Potential implications for age-related macular degeneration in Alzheimer’s dementia

**DOI:** 10.3389/fnagi.2022.1015359

**Published:** 2022-11-16

**Authors:** Bijorn Omar Balzamino, Graziana Esposito, Ramona Marino, Pietro Calissano, Valentina Latina, Giuseppina Amadoro, Flavio Keller, Andrea Cacciamani, Alessandra Micera

**Affiliations:** ^1^Research and Development Laboratory for Biochemical, Molecular and Cellular Applications in Ophthalmological Sciences, IRCCS – Fondazione Bietti, Rome, Italy; ^2^Laboratory of Developmental Neuroscience and Neural Plasticity, Campus Bio-Medico University, Rome, Italy; ^3^European Brain Research Institute (EBRI), Rome, Italy; ^4^Institute of Translational Pharmacology (IFT)-CNR, Rome, Italy

**Keywords:** Reelin (RELN), inflammatory/anti-inflammatory factors, Alzheimer’s disease (AD), age-related macular degeneration (AMD), vitreous, retinal disease

## Abstract

The neurosensory retina is an outgrowth of the Central Nervous System (CNS), and the eye is considered “a window to the brain.” Reelin glycoprotein is directly involved in neurodevelopment, in synaptic plasticity, learning and memory. Consequently, abnormal Reelin signaling has been associated with brain neurodegeneration but its contributing role in ocular degeneration is still poorly explored. To this aim, experimental procedures were assayed on vitreous or retinas obtained from Reeler mice (knockout for Reelin protein) at different postnatal days (p) p14, p21 and p28. At p28, a significant increase in the expression of Amyloid Precursor Protein (APP) and its amyloidogenic peptide (Aβ1-42 along with truncated tau fragment (i.e., NH_2_htau)- three pathological hallmarks of Alzheimer’s disease (AD)-were found in Reeler mice when compared to their age-matched wild-type controls. Likewise, several inflammatory mediators, such as Interleukins, or crucial biomarkers of oxidative stress were also found to be upregulated in Reeler mice by using different techniques such as ELLA assay, microchip array or real-time PCR. Taken together, these findings suggest that a dysfunctional Reelin signaling enables the expression of key pathological features which are classically associated with AD neurodegenerative processes. Thus, this work suggests that Reeler mouse might be a suitable animal model to study not only the pathophysiology of developmental processes but also several neurodegenerative diseases, such as AD and Age-related Macular Degeneration (AMD), characterized by accumulation of APP and/or Aβ1-42, NH_2_htau and inflammatory markers.

## Introduction

The eye is considered as “a window to the brain” since the neuroretina is more accessible than brain for non-invasive imaging (Chu et al., 2012). In this context, different research studies have been focused on the application of ophthalmic diagnostic procedures for the clinical management of Alzheimer disease (AD) (Javaid et al., 2016; Mirzaei et al., 2020). Loss in synapses, amyloid β (Aβ) peptide aggregation and accumulation of tau neurofibrillary tangles into specific Central Nervous System (CNS) regions are mainly required for AD diagnosis (Galasko and Shaw, 2017; Jack et al., 2017). An early accumulation of APP/Aβ and tau hyperphosphorylation, two hallmarks of brain neurodegeneration, also occur in retinas of experimental AD models and patients suffering from AD (Oddo et al., 2003; Javaid et al., 2016; Czakó et al., 2020; Mei et al., 2020; Schultz et al., 2020; Gupta et al., 2021; Latina et al., 2021a). Additionally, AD patients state visual deficits and retinal ultrastructural modifications, such as ganglion cell degeneration, nerve fiber layer (NFL) thinning and optic nerve degeneration (Koronyo-Hamaoui et al., 2011; Schultz et al., 2020), supporting the notion that the retina embodies a valuable site for preclinical level of AD biomarkers research (Cordeiro et al., 2010). Other key features in the pathophysiology of AD eye are inflammation (Cameron and Landreth, 2010; Calsolaro and Edison, 2016) and microglia over-activation that cause damage to the Ganglion Cell Layer (GCL) and Inner Nuclear Layer (INL) (Eriksson et al., 1998; Lugert et al., 2010). Interestingly and more importantly, AD shares several common features with an ocular disease known as age-related macular degeneration (AMD), including retinal Aβ deposition (Platania et al., 2017; Micera et al., 2019; Ashok et al., 2020; Amadoro et al., 2021).

Reelin is a glycoprotein that plays a key role in neuronal survival, differentiation, synaptic plasticity, and memory development (Balzamino et al., 2014, 2019; Hirota et al., 2015). Altered Reelin signaling is detected in human brain samples of AD patients (Notter and Knuesel, 2013; Pujadas et al., 2014), in correlation with APP/Aβ deposition (Pujadas et al., 2014) and tau phosphorylation (Hoe et al., 2009; Cuchillo-Ibáñez et al., 2013; Rice et al., 2013). Decreased levels of brain Reelin are associated with altered synaptic plasticity, cytoskeleton instability and axonal transport impairments (Krstic et al., 2013; Yu et al., 2016). Some experimental models have displayed that Reelin protects from the deleterious effects of APP deposits (Durakoglugil et al., 2009). As observed in a transgenic AD Mice model, APP/Aβ plaque occurs in concomitance of Reelin reduction in some specific regions of the brain (Niu et al., 2008; Kocherhans et al., 2010). The main aspects of Reelin activity in the visual system encompass retinogenesis, inflammation and tissue remodeling (Rice et al., 2001; Micera et al., 2016). Although the biological action of Reelin has been largely depicted in the brain, the potential contributing role in ocular degeneration remains poorly investigated (Ashok et al., 2020). Therefore, our working hypothesis is that a deficient Reelin signal inside the retinal network might lead to the expression of APP/Aβ and NH_2_htau alongside a robust neuroinflammation.

The main aim of our study carried out on Reelin retinas was to (i) assess the presence of APP/Aβ and pathological NH_2_htau and (ii) verify the presence of some (neuro)inflammatory mediators frequently reported in association with AD biomarkers.

## Materials and methods

### Animals and ethical approval

Thirty-six (36) animals were used for the study, including eighteen (18) Reeler (male Reeler-L7-EGFPreln-/- strain; 9–11 g body weight) and 18 WT (male B6C3Fe-L7-EGFPreln+/+ strain; 12–14 g body weight) mice (Charles River, Calco, Como). Reeler (B6C3Fe-a/a-rl in a C57BL/6J background) and WT mice were at postnatal day (p) p14, p21 and p28. Animals were divided by assay: 18 animals (9 Reeler and 9 WT) for Confocal analysis, divided in subgroups: 3 Reelers for time point p14, p21 and p28, and 3 WT for time point at p14, at p21 and at p28; and 18 animals (9 Reeler and 9 WT) for all other assays. Both eyes were used, and the overall experimental procedure was summarized in Supplementary Figure 1.

Experimental procedures were approved by the Ethical Committee of Tor Vergata University (Rome, Italy) and the Ethical Committee of Campus Biomedico University (Rome, Italy), according with ethical standards stated in the Declaration of Helsinki and the ARVO Statement for the Use of Animals in Ophthalmic and Vision Research. All the steps in the procedure were in compliance with the directive of 2010/63/EU guidelines, under the authorization provided by the Italian Ministry of Health. All efforts were made to reduce number and minimize suffering.

### Experimental procedure: Vitreous and retina

Mice were anaesthetized by intraperitoneal injection of 2 mg/ml ketamine (0.2 ml/10 g body weight; Ketavet, Gellini Pharmaceutics, Italy) and 0.23 mg/ml medetomidine (0.24 ml/10 g body weight; Domitor, Orion Corp., Espoo, Finland) mixture. Vitreous and retinas were dissected out under a stereomicroscope (SMZ645; Nikon, Tokyo, Japan) equipped with cold-light optic fibers (PL2000 photonic; Axon, Vienna, Austria), according to our standard procedure (Balzamino et al., 2015). Briefly, a corneal incision was generated and retina and vitreous were collected in microvials equipped with membranes suitable for centrifugation (13,000 rpm/15 min), to detach vitreous from retina. Vitreous and retina were appropriately stored for biochemical and molecular studies.

Not-pooled samples were, respectively stored as untouched for vitreous (biomolecular assays) and post-fixed and paraffin embedded for retinas (imaging analysis). Tissue extractions were performed in modified RIPA Buffer (50 mM Tris–HCl, 150 mM NaCl, 1% Triton-X100, 5 mM EDTA, 100 mM NaF and 1 mM PMSF; pH 7.5) for biochemical assay or TRIfast solution for molecular analysis, according to standard procedures (Balzamino et al., 2019). Total proteins or total RNAs were quantified with microvolume Spectrophotometer (Nanodrop N1000, Celbio, Milan, Italy).

### Confocal analysis

Paraffine-embedded retinas (9 Reeler and 9 WT; 1 retina = 5 slides = 3 optic fields per slide) were sectioned and subjected to double-immunofluorescence. Briefly, dewaxed and post-fixed (2% buffered ρ-Formaldehyde; PFA) sections were equilibrated in PBS [10 mM phosphate buffer and 137 mM NaCl; pH 7.5], quenched (10 mM NH_4_Cl), permeabilized (0.5% Triton X100 in PBS; PBS-TX) and probed with the following antibodies: anti-mouse APP (B4) (sc-28365; 1/100; Santa Cruz Biotechnology; Dallas, Texas, USA); anti-rabbit Aβ1-42 peptide (D54D2) (mAb #8243; 1:100; Cell Signalling Technology, Inc.; Danvers, Massachusetts, USA); anti-rabbit caspase-cleaved protein (CCP)-NH_2_ tau 4268; 1/200 Amadoro et al., 2012; Latina et al., 2021a,b) and anti-rabbit TLR4 (H-80) antibody (sc-10741; 1/100; Santa Cruz) as shown in Table 1A. The specific binding was detected using Cy2/Cy3-conjugated specie-specific secondary antibodies (1/500-1/700; Jackson ImmunoResearch Labs., Europe Ltd., Suffolk, UK). Nuclei were counterstained with DAPI (5 μg/ml; Invitrogen-Molecular Probes, Eugene, Oregon). Acquisitions were carried out using the TE2000U confocal microscope equipped with C1 software (Nikon, Tokyo, Japan). Internal control sections were provided by substituting the primary antibody with control irrelevant IgGs (Vector Laboratories, Inc., Burlingame, CA) and were used for channel-series setup (Nikon). An unbiased approach was used for all quantitative analyses. Integrated Optical Density (IntDen) signals were quantified by the free available ImageJ v1.43 software (NIH-http://rsb.info.nih.gov/ij/). Digital images and graph-plot were assembled by using Adobe Photoshop 2022 program (Adobe Systems Inc., San Jose, CA).

**TABLE 1:** **(A)** Immunoprecipitation/immunofluorescence. **(B)** Molecular analysis.

(A)				
Target	Dilution	Host	Specificity	Source
Anti-APP	1:100	Mo	Regulator of synapse formation and neural plasticity	Santa cruz
Anti-Aβ1-42	1:100	Rb	Main component of amyloid plaques deposits	Cell Signaling
Anti-NH_2_htau	1:100	Rb	Modulate the stability of axonal microtubules (caspase-cleaved protein (CCP)-NH_2_ tau 4268)	Amadoro et al., 2012
Anti-MMP9	1:100	Mo	Cleaves the extracellular matrix	Santa cruz
Anti-IL6	1:100	Mo	Pro-inflammatory cytokine	Santa cruz
Anti-TLR4	1:100	Rb	Role in pathogen recognition and innate immunity	Santa cruz

**(B)**				
**Target**			**Sequence primers**	**Tm/Amplicon**

*APP*			5′-GGA GCC CAC CAA GAA CGA T-3′ 3′-TCA CCA GCA TCA GTC CCA AG-5′	60°C/162 bps
*TAU*			5′-TAG CAA CGT CCA GTC CAA GT-3′ 3′-TTC CCT AAC GAG CCA CAG TT-5′	57°C/185 bps
*IL6*			5′-GGA GCC CAC CAA GAA CGA T-3′ 3′-TCA CCA GCA TCA GTC CCA AG-5′	59°C/100 bps
*IL8R*			5′-TCT CTT GGA AGC CTT CTT G-3′ 3′-TGG GGT GGA AAG GTT TGG-5′	58°C/100 bps
*IL18*			5′-CTT TGG CCG ACT TCA CTG TAC A-3′ 3′-GGG GTT CAC TGG CAC TTT GAT-5′	60°C/125 bps
*iNOS*			5′-CCC CTT CAA TGG CTG GTA CA-3′ 3′-GTT TCC AGG CCC ATT CTC CT-5′	59°C/100 bps
*NOX4*			5′-CTC AGC GGA ATC AAT CAG CTG TG-3′ 3′-AGA GGA ACA CGA CAA TCA GCC TTA G-5′	62°C/100 bps
*NRF2*			5′-ACA CGG TCC ACA GCT CAT C-3′ 3′-TGC CTC CAA AGT ATG TCA ATC A-5′	58°C/100 bps
*KEAP1*			5′-GGG TCC CCC CTA CAG CCA AG-3′ 3′-TGG GGT TCC AGAAGA TAA GC-5′	59°C/100 bps
*HDAC1*			5′-GTG GTT CTG TGG CAA GTG C-3′ 3′-TGT ACA GCA CCC TCT GGT GA-5′	56°C/117 bps
*DNMT3a*			5′-GCA CTC AAG GGC AGC AGA TA-3′ 3′-TTC CAG GCT TCC CAG GGT TAG-5′	59°C/100 bps
*TLR4*			5′-ATT CCC CTG AGG CAT TTA GG-3′ 3′-CAG GGC TAA ACT CYG GAT GG-5′	60°C/201 bps
*GAPDH*			5′-GTG GAC CTC ATG GCC TAC AT-3′ 3′-GTT GGG ATA GGG ACT CCT CAC-5′	60°C/100 bps

Amplification profile: hot start activation (95°C/5 min); 39 cycles: den. at 94°C/10s – ann. at 56–61°C/30 sec – ext. at 72°C/15 sec; melting curve recording 55–95°C with one fluorescence reading every 0.5°C.

### Reverse transcription real-time PCR

Total RNA extraction was performed with TRIfast solution, according to the manufactures’ procedure (EuroClone, PV, Italy). Equivalent total RNAs (30 ng/sample; 260/280 > 1.8; Nanodrop) were used for cDNA synthesis in the presence of 50 pM random primers (IMPROM kit, Promega, Milan, Italy) in a programmable PCR thermocycler (Peqlab LLC., Wilmington, USA). SYBR Green PCR amplifications (Applied Biosystems, Foster City, CA) were run in Eco Illumina PCR platform (Illumina Inc., San Diego, CA, USA). Negative and positive controls were run in parallel. Cq values were automatically generated from normalized samples showing one melting curve. Changes in transcript expression were provided as 2log expression ratio of Reeler with respect to WT (used as referring group), considering *GAPDH* as referring gene (REST software, Pfaffl, 2001). Primer pairs were designed one intron spanning (https://www.ncbi.nlm.nih.gov and http://primer3.ut.ee) and synthesized by Eurofin MWG Genomics (www.eurofinsgenomics.eu). The specific primers sequences (*GAPDH, APP, TAU, IL6, IL8R, IL18, TLR4, iNOS, NOX4, NRF2, KEAP1, HDAC1* and *DNMT3a*) and the amplification program are shown in Table 1B. Negative/positive controls and single-mode melting curves were used to confirm the amplifications.

### Ella™ microfluidics-based platform

The multiplex Ella™ platform was used to quantify the specific expression of Interleukin 6 (IL6), IL8R, IL18, Matrix Metalloproteinase 9 (MMP9), Osteopontin (OPN) and Cysteine-rich angiogenic inducer 61 (CYR61) in retinal extracts and vitreous samples. Briefly, 10 μl samples were diluted 1:3 in assay buffer and added to cartridge, according to a standard procedure provided by the manufacturers (Protein Simple, CA, USA). All steps in the immunoassay procedure were conducted automatically. Cartridges included build-in lot specific standard curve and samples were run as internal triplicates. Single data for each sample were automatically calculated and expressed as pg/ml.

Changes in transcript expression were provided as 2logFC expression ratio of Reeler with respect to WT (used as referring group), considering GAPDH as referring gene (REST software, Pfaffl, 2001). Primer pairs were designed one intron spanning (https://www.ncbi.nlm.nih.gov and http://primer3.ut.ee) and synthesized by Eurofin MWG Genomics (www.eurofinsgenomics.eu). The specific primers sequences (*GAPDH, NH_2_htau, IL6, IL8R, IL18, TLR4, iNOS, NOX4, NRF2, KEAP1, HDAC1 and DNMT3a*) and the related amplification program are shown in Table 1B.

### Immunoprecipitation and western blot analysis

Magnetic Beads (Protein A Magnetic Beads; Thermo Scientific Pierce, Waltham, MA, USA) were used for immunoprecipitation of specific proteins using affinity binding antibodies (Jensen et al., 2021). Briefly, prewashed beads were conjugated with specific antibodies in PBS with 0.05% of Tween (PBST) (50 μl beads and 5 μl of antibody): anti-mouse MMP9 (E-11; sc-393859; Santa Cruz); anti-mouse IL6 (10E5; sc-57315; Santa Cruz); anti-rabbit TLR4; anti-rabbit NH_2_htau and anti-mouse β-Actin (C4; sc-47778; Santa Cruz). Antibody-bead complex was performed at room temperature under gentle orbital shaking (Vélez-Bermúdez et al., 2022) and after 30 min the complex was cleaned up with PBST and added to 50 μg of total protein lysate for 1 h of incubation. Finally, the specific antibody-beads-protein complexes were eluted in 2x Loading-Buffer (Invitrogen) supplemented with β-mercaptoethanol, boiled (98°C/5 min) and electrophoresed in 4–20% SDS-PAGE minigels (miniprotean; Biorad, Hercules, California, USA). After separation, gels were stained according to a standard protocol (SYPRO Ruby gel stain; Thermo Fisher, Massachusetts, USA) and acquired in a B-BOX Blue Light LED epi-Illuminator (Smobio, Hsinchu City, Taiwan). Band analysis was performed by using ImageJ v1.43.

### Chip array analysis

Inflammatory/profibrogenic factors were quantified in vitreal samples by a customized chip-based array, among a list of potential candidates (G-series arrays; Ray Biotech, Norcross, CA, USA). Each glass-slide chip consisted of 14 identical sub-arrays, with 50 biomarkers (antibody spots in duplicate) with hindsight selected by bibliography research. Reeler and WT samples were processed simultaneously. Briefly, normalized vitreous (30 μg total protein ≈ 25 μl per well) were diluted in proper buffer, for a total of 70 μl per sample, and hybridized as described in the manufacturer’s protocol including wash, detection and label steps. Spin-dried slides were scanned in a GenePix 4400 Microarray platform (Molecular Devices LLC, Sunnyvale, Silicon-Valley, CA, USA). Capturing conditions and image digital acquisitions were done as previously reported (Balzamino et al., 2019). In addition to being uniformly adjusted for size, brightness, contrast, and chip-to-chip comparisons by the software, the images were also provided to the user as 8-bit Tiff files (Axon GenePix Pro 6.0 software: Molecular Devices). Several internal controls were present for each sub-array to ensure inter-assay normalization. The sensitivity range was 3.8–56 pg/ml, as provided by the manufacturer.

### Statistical analysis and repository actions

The data analyses were performed using the GraphPad Prism 9.4 software (GraphPad Software; San Diego, CA, USA). Lab parameters were tested for normal distribution using a Shapiro–Wilk test. According to the Shapiro–Wilk test, the comparisons between the biomarkers’ levels were performed using the Kruskal-Wallis test with Dunn’s correction for multiple comparisons. For Integrated Optical Density (IntDen), the 8-bit TIFF saved digital images (512 × 512 or 1,024 × 1,024 dpi; *n* = 5 sections/slide; x40/dry 0.75 DIC M/N2) were subjected to single analysis with the ImageJ. IntDen data (mean ± SD/optic field) were calculated, grouped, and subjected to statistical analysis. Significance between groups was set at *p < 0.05, ^**^p < 0.01 and ^***^p < 0.001. For array results, data were deposited in a public repository (ArrayExpress) with a provisional accession number: *E-MTAB-7622* (14/01/2019), until the definitive acceptance of the study protocol in the ArrayExpress platform. Significance between groups was estimated by using the two-sided unpaired *t*-test statistical comparisons with Bonferroni corrections for number of targets (^***^p < 0.001; *p* = 0.05/50 targets). Results are shown as mean ± standard errors of the mean (SEM) in the bar-graph, while the Standard Deviation was reported in the text.

## Results

### Increased APP/Aβ1-42 and pathological tau truncation (NH_2_htau) AD-like immunoreactivities are detected in retinas of Reeler mice

To assess the presence of classical AD-like neuropathological hallmarks in Reeler eyes, the expression profile of APP, Aβ1-42 and pathological NH_2_htau cleaved form were investigated by immunofluorescence studies on serial retinal sections from adult animals at p28. To this aim, we used the B-4 -a commercial anti-APP specific antibody (aa 672-714 epitope)- the D54D2 -another commercial antibody selective for the Aβ1-42 peptide- and the cleavage-specific monoclonal NH_2_htau antibody (D_25_-(QGGYTMHQDQ) epitope, phosphorylation-independent state (Corsetti et al., 2020; Latina et al., 2021b) which selectively detects the neurotoxic AD-relevant 20–22 kDa fragment (NH_2_htau), both in brain and in eye.

**FIGURE 1:**
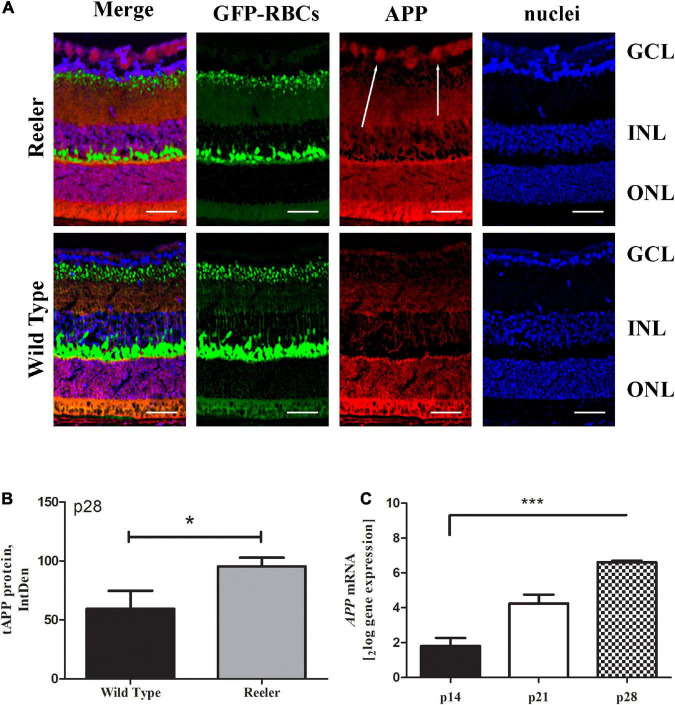
Total APP immunoreactivity in Reeler retinas. **(A)** Representative confocal fluorescence microscopy images showing GFP-expressing RBCs (green), total APP immunoreactivity (red) and nuclear staining (dapi/blue). White arrows point to a noticeable staining of APP (red) at the GCL of Reeler retinas. **(B)** Histogram representative of APP IntDen analysis (mean ± SEM) in both Reeler and WT retinas. Note the increased values for APP in p28 Reeler with respect to WT (*n* = 3 optic fields per slide of 3 animals per group; *p < 0.05, Kruskal Wallis analysis with post Dunn’s multiple comparison test analysis). **(C)** The mRNA expression levels of *APP* was assessed in not pooled retinas derived from Reeler and WT mice at p14, p21 and p28 days of age, normalized with *GAPDH* and shown as fold changes (***p < 0.0001; REST analysis). APP, transmembrane Amyloid Precursor Protein; GFP, Green Fluorescent Protein; RBCs, Rod Bipolar Cells; GCL, Ganglion Cell Layer; INL, Inner Nuclear Layer; ONL, Outer Nuclear Layer; IntDen, Integrated Optical Densitometry. Magnifications: ×40; white bar: 20 μm.

As shown in Figure 1A, an increased diffuse labeling for total APP (red) – which was mainly confined at the GCL and pointed by arrows – was clearly observed in Reeler retinas when compared to their age-matched WT controls. The immunoreactivity was corroborated by the densitometric analysis carried out on single images (tAPP at p28; 1 retina = 5 slides = 3 optic fields per slide), as shown in the histogram (75.50 ± 7.64 *vs.* 45.52 ± 20.08 IntDen; *p* < 0.05, Reeler *vs.* WT; Figure 1B). Real time PCR at all time points confirmed this upregulation for *APPmRNA* (p14: 1.80 ± 0.13_2*log–ratio*_; p21: 4.25 ± 0.14_2*log–ratio*_; p28: 6.60 ± 0.04_2*log–ratio*_; *p* < 0.0001; REST analysis, Reeler *vs.* WT; Figure 1C).

**FIGURE 2:**
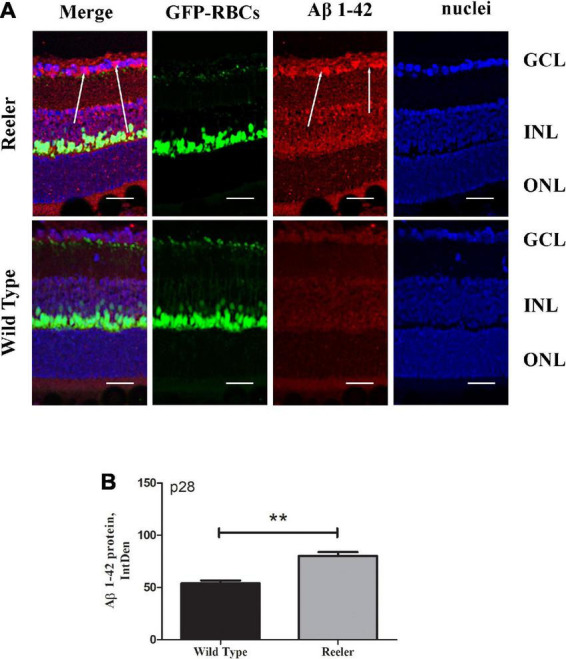
Retinal expression of Aβ1-42 increase in Reeler p28 mice. **(A)** Immunofluorescent images for Aβ1-42 (red), GFP (green), and nuclei (blue) in retina of p28 mice. White arrows point to a noticeable staining of Aβ1-42 (red) at the GCL of Reeler retinas. **(B)** IntDen of Aβ1-42 -positive cells was assessed (*n* = 3 optic fields per slide of 3 animals per group; **p < 0.01, Kruskal Wallis analysis with post Dunn’s multiple comparison test analysis). Note the increased expression of Aβ1-42 oligomers (mean ± SEM) in Reeler mice with respect to WT. Aβ1-42, Amyloid β Protein Fragment 1-42; GFP, Green Fluorescent Protein; GCL, Ganglion Cell Layer; INL, Inner Nuclear Layer; ONL, Outer Nuclear Layer; IntDen, Integrated Optical Densitometry. Magnifications: ×40; white bar: 20 μm.

To corroborate the APP expression, we investigated whether the toxic Aβ1-42 oligomers were expressed, to the same cellular compartments of APP, in the retinal Ganglion Cell Layers (GCLs) of aged mice. (Figure 2A). High levels of Aβ1-42 oligomers localized in ganglion neurons of reeler mice with respect to WT ones. This accumulation of both total APP and Aβ1-42 oligomer suggests, according to human AD, that they may function synergistically to exacerbate synaptic dysfunction and neuronal death. Aβ1-42 densitometric analysis (80.24 ± 7.04 *vs.* 53.96 ± 5.33 IntDen; *p* < 0.01, Reeler *vs.* WT; Figure 2B).

**FIGURE 3:**
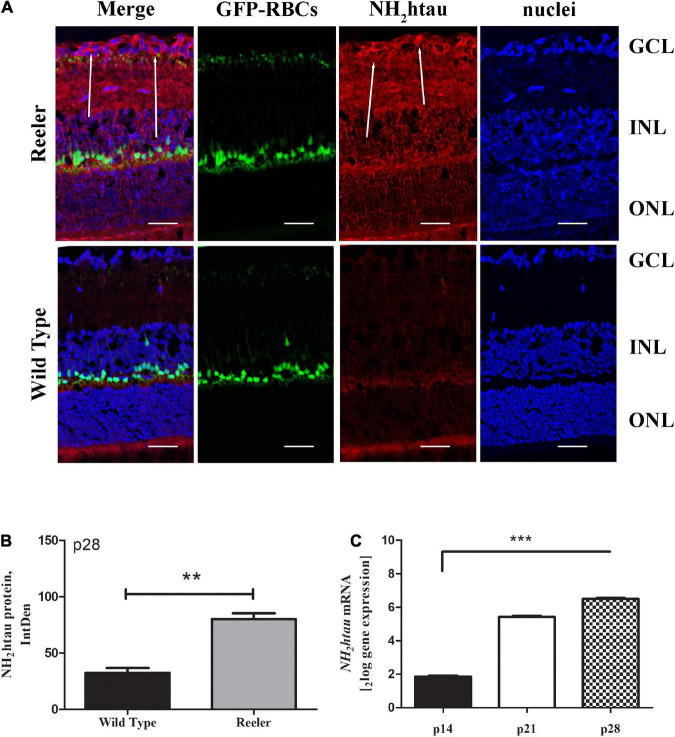
The NH_2_htau expression in Reeler retinas. **(A)** Representative confocal fluorescence microscopy images (p28) showing NH_2_htau immunoreactivity and GFP-expressing RBCs (merge; green/red) over a nuclear staining (dapi/blue), and single-channel acquisitions (t-NH_2_htau/red; GFP-expressing RBCs/green; dapi/blue). White arrows point to a noticeable staining of NH_2_htau (red) at the GCL of Reeler retinas. **(B)** Histogram representative of NH_2_htau IntDen analysis in both Reeler and WT retinas (p28). Note the increased expression of NH_2_htau protein (mean ± SEM) in Reeler mice with respect to WT (*n* = 3 optic fields per slide of 3 animals per group; **p < 0.01, Kruskal Wallis analysis with post Dunn’s multiple comparison test analysis). **(C)** Retina extracts confirmed the presence of *tau* transcript starting from p21 (***p < 0.0001, REST analysis). NH_2_htau, truncated tau; GFP, Green Fluorescent Protein; RBCs, Rod Bipolar Cells; GCL, Ganglion Cell Layer; INL, Inner Nuclear Layer; ONL, Outer Nuclear Layer; IntDen, Integrated Optical Densitometry. Magnifications: ×40; white bar: 20 μm.

Likewise, the NH_2_htau immunoreactivity appeared to be significantly increased in p28 Reeler retinas in comparison with controls (Figure 3A). This increased expression of truncated NH_2_htau protein (80.00 ± 10.52 *vs.* 32.43 ± 8.83 IntDen; *p* < 0.01, Reeler *vs.* WT) was quantified by IntDen analysis (p28; Figure 3B), also corroborated by molecular data highlighting the upregulation of *TAU* transcript as soon as p21 (p14: 1.82 ± 0.03_2*log–ratio*_; p21: 5.39 ± 0.04_2*log–ratio*_; p28: 6.46 ± 0.08_2*log–ratio*_; *p* < 0.0001, p21 and p28; REST analysis; Reeler *vs.* WT; Figure 3C).

### Increased inflammatory, oxidative stress inducers and matrix enzymes expression are found in the retinas of Reeler mice in correlation with high load of total NH_2_htau protein

**FIGURE 4:**
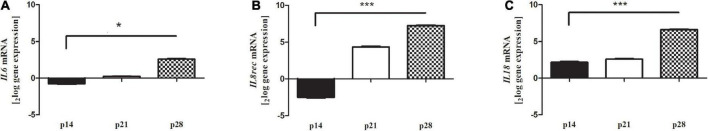
**(A)** *IL6*, **(B)** *IL8R* and **(C)** *IL18* transcript expression in retinal extracts. Total RNA was used to generate cDNA for real time PCR analysis. Histograms show a significant upregulation at p28 for all targets investigated. Significant differences are shown as *p < 0.01, ***p < 0.0005, REST analysis; Reeler *vs*. WT). Data are 2logFC transcript expression (mean ± SEM, Reeler *vs.* WT).

Real-time PCR experiments were conducted to analyze the expression of several inflammatory mediators. As shown in Figure 4, we observed a significant increase of *IL6, IL8R* and *IL18* transcripts, specifically at p28, in retinas from Reeler mice as compared to controls (**A:** *IL6*: 2.59 ± 0.06_2*log–ratio*_, *p* < 0.01; **B:** *IL8R*: 7.24 ± 0.15_2*log–ratio*_, *p* < 0.0001; **C:** *IL18:* 6.59 ± 0.09_2*log–ratio*_, *p* < 0.0001; REST analysis; all Reeler *vs.* WT).

**FIGURE 5:**
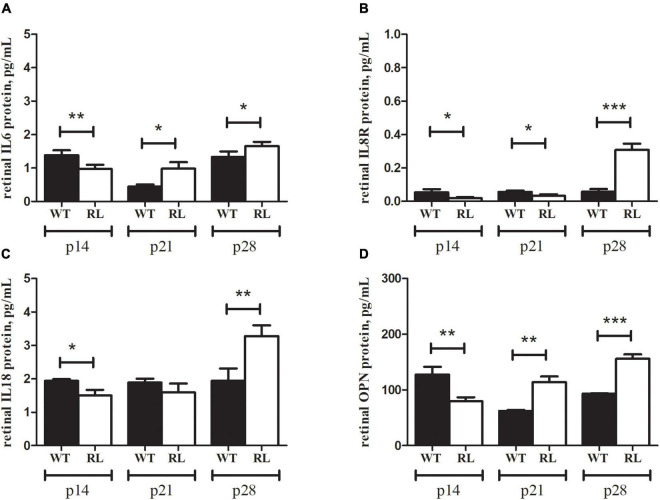
IL6, IL8R, IL18 and OPN signature in Reeler retina. Ella microfluidic analysis was performed on prediluted retinal extracts from Reeler (RL) and WT mice. Bar-plot graphics show the individual values for **(A)** IL6, **(B)** IL8R, **(C)** IL18 and **(D)** OPN. Note that all biomarkers show a high significance at p28 (p28; RL *vs.* WT; two tailed unpaired t test with Welch’s correction). While a significant down regulation was observed for all markers at p14 (p14; RL *vs.* WT; Kruskal Wallis analysis with post Dunn’s multiple comparison test analysis) in Reeler mice. Specific *p* values are shown by asterisks (*p < 0.05; **p < 0.01 or ***p < 0.005).

Comparable results were obtained by ELLA microfluidic assay on retinal extracts (Figure 5). Particularly, IL6, IL8R and IL18 were upregulated at p28, as compared to WT ones. Increased protein levels were observed for IL6, IL8R and IL18 specifically at p28 Reeler *vs.* WT (**A**, **B** and **C**). Of interest, the low levels of OPN protein detected in Reeler extracts at p14 (*p* < 0.003) turned out high at p21 (*p* < 0.002) and p28 (*p* < 0.0005) (**D**). Ella assay confirmed similar CYR61 protein expression at all time points investigated (histogram not shown).

**FIGURE 6:**
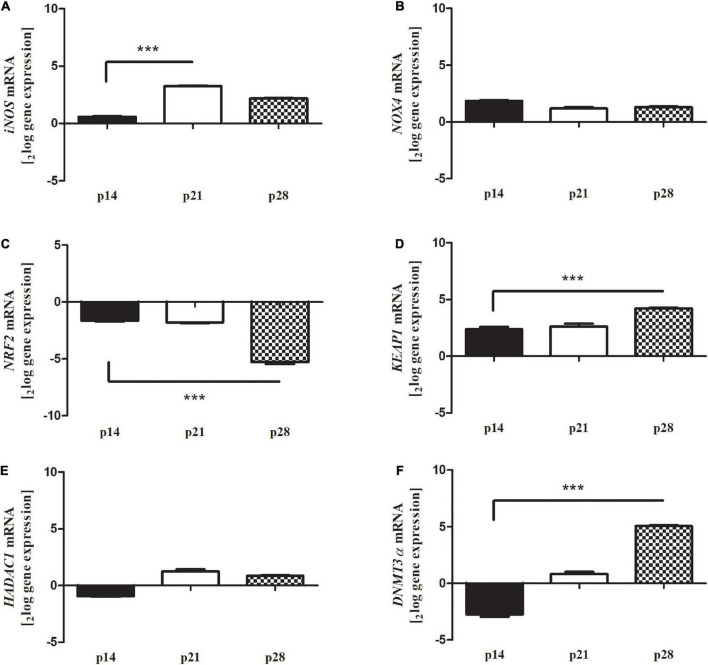
Differential expression of oxidative stress markers in Reeler retina. Total RNA was extracted from non-pooled retinas and used to generate cDNA for real time PCR analysis. Histograms show a significant transcript upregulation for oxidative stress markers [**(A)** *iNOS*, **(D)** *KEAP1* and **(F)** *DNMT3a*] and a significant downregulation of **(C)** *NRF2* at p28 in Reeler retinas as compared to WT ones; REST analysis). No significant changes were detected for **(B)** *NOX4* and **(E)** *HDAC1*. Data are 2logFC gene expression (mean ± SEM, Reeler *vs.* WT) and *p* values are shown by asterisks (***p < 0.0001).

In addition, oxidative stress modulation was assessed by real-time PCR experiments, analyzing the involvement and the expression of well-known stressor transcripts (Figure 6). The amplification showed an increased expression for *iNOS* at p21 (**A**, *p* < 0.0001, 3.26 ± 0.09_2*log–ratio*_); unchanged expressions for *NOX4* at p28 (**B**, *p* > 0.05); a significant deregulation for *NRF2* at p28 (**C**, *p* < 0.0001, -5.41 ± 0.02_2*log–ratio*_); a significant upregulation for *KEAP1* at p28 (**D**, *p* < 0.0005; 4.23 ± 0.18_2*log–ratio*_), unchanged expressions for *HADAC1* (**E**, *p* > 0.05) and a significant upregulation for *DNMT3*α at p28 (**F**, *p* < 0.0001, 5.08 ± 0.03_2*log–ratio*_). All changes were obtained according to the REST analysis, comparing Reeler *vs.* WT retinas’ specific amplifications. Of interest, *NOX4* and *HDAC1* showed no changes at each time-point investigated.

**FIGURE 7:**
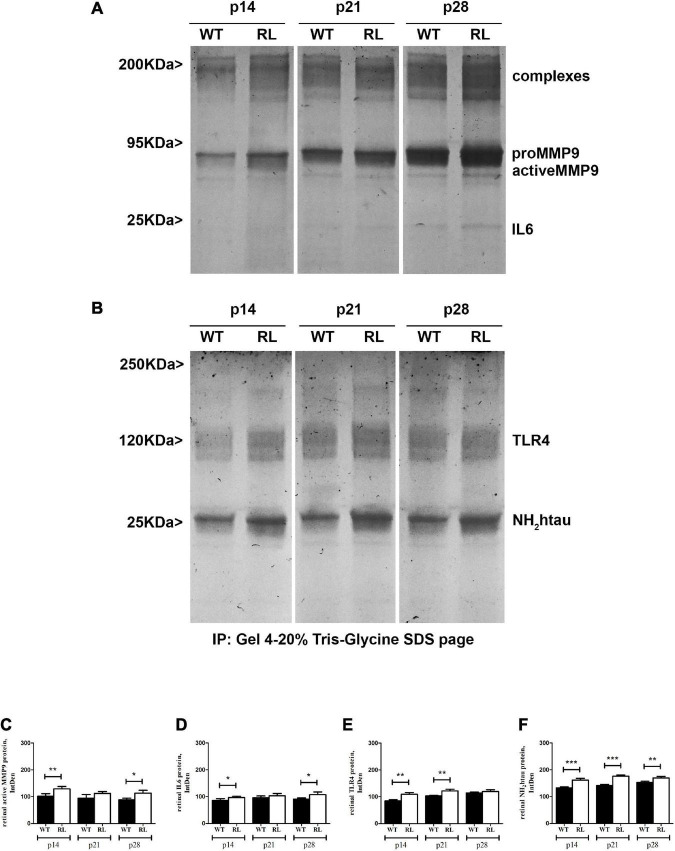
Representative immunoblots (4–20%) and relative densitometric analysis of control (WT) and Reeler (RL) retinal protein extracts, as probed and quantified: MMP9 **(A,C)**; IL6 **(A,D)**; TLR4 **(B,E)** and NH_2_htau **(B,F)** antibodies (IntDen values; *p < 0.05, **p < 0.01, ***p < 0.005; Kruskal Wallis analysis with post Dunn’s multiple comparison test analysis). The proteins were normalized against β-actin; the original re-probed β-actin immunoblot is shown in Supplementary Figure 2.

MMP9, IL6, TLR4 and NH_2_tau proteins were differentially expressed in Reeler retinas as compared to WT, as shown by immunoblots (IP/WB) and related band analysis (IntDen). As quantified (Figure 7A), the increase of proMMP9/activeMMP9 ratio occurred in a time-dependent fashion (see band, 95 KDa), while IL6 protein increased slightly at p28 (see band, 25 KDa). While NH_2_htau, in retinas from Reeler mice, increased at all stages analyzed when compared to WT ones (Figure 7B). Though TLR4 seems upregulated at early stages (p14 and p21; *p* < 0.001) and no significant changes are visible at p28 (*p* > 0.05). The related band quantifications are shown, respectively in Figures 7C–F.

**FIGURE 8:**
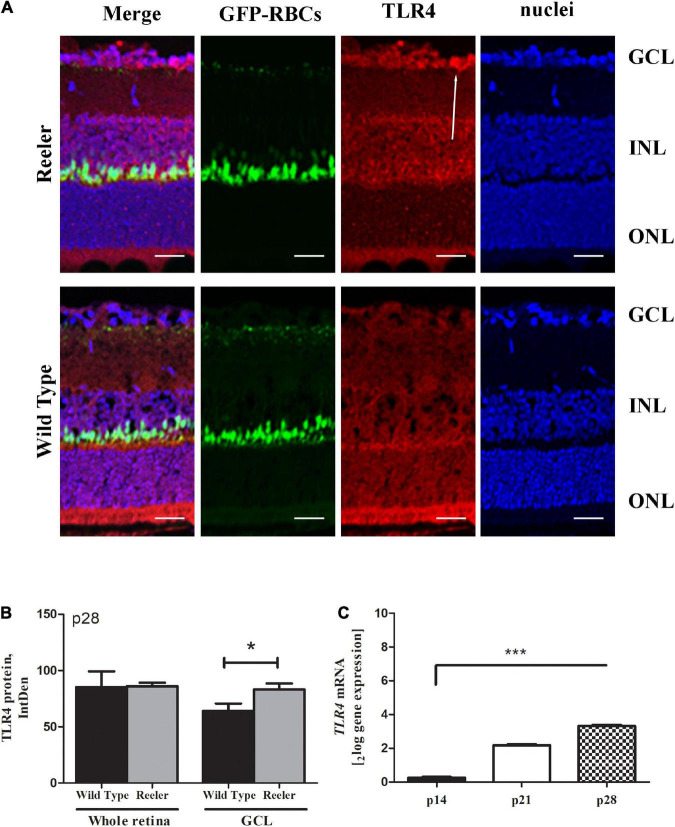
TLR4 expression in Reeler retinas. **(A)** Representative illustrations (confocal fluorescence microscopy) showing TLR4 immunoreactivity (red) in GFP-expressing RBCs (green) and nuclear (blue) retinal sections (merge). Single-channel acquisitions are TLR4/red; GFP-expressing RBCs/green and nuclei/dapi/blue. White arrows point to a noticeable immunoreactivity of TLR4 (red) in the GCL zone of Reeler retina. **(B)** Histogram representative of TLR4 IntDen quantifications (mean ± SEM; Reeler *vs*. WT, (*p* > 0.05) in whole retinas and (*p < 0.05; Kruskal Wallis analysis with post Dunn’s multiple comparison test analysis) in the GCL cell compartment). **(C)** mRNA retinal extracts highlighted the *TLR4* transcript upregulation at p28 (***p < 0.0001, REST analysis). Note the increased values for *TLR4* in Reeler with respect to WT. TLR4, Toll-like Receptor 4; GFP, Green Fluorescent Protein; RBCs, Rod Bipolar Cells; GCL, Ganglion Cell Layer; INL, Inner Nuclear Layer; ONL, Outer Nuclear Layer; IntDen, Integrated Optical Densitometry. Magnifications: ×40; white bar: 20 μm.

Immunofluorescence studies (Figure 8A) showed a non-significant TLR4 immunoreactivity in whole retinal sections (86.07 ± 6.12 *vs*. 85.25 ± 27.78 IntDen; *p* > 0.05, Reeler *vs*. WT; Figure 8B), while a significant upregulation at the GCL of p28 Reeler mice as compared to WT ones (83.23 ± 10.50 *vs*. 64.02 ± 13.24 IntDen; *p* < 0.02, Reeler *vs*. WT; Figure 8B). It’s noteworthy to highlight the specific immunoreactivity of TLR4 at the GCL, as pointed out by the white arrow. As for REST analysis, *TLR4* transcripts were specifically upregulated at p28 (*p* < 0.0001; 3.24 ± 0.09_2*log–ratio*_, Figure 8C).

**FIGURE 9:**
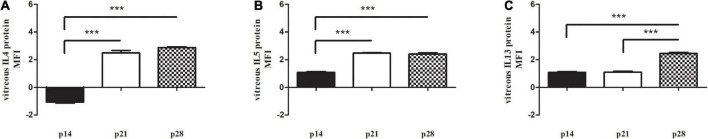
Vitreal signature in Reeler mice. Chip array analysis was performed on prediluted vitreal samples from Reeler (RL) and WT mice. Bar plots representative of modulators of **(A)** IL4, **(B)** IL5 and **(C)** IL13 proteins, as detected by Axon software after fluorescence acquisition (Genepix) at p14, p21 and p28, as compared to WT. Note the selective increased expression at p28. Fold changes [± 2 FC, from data supplied as Mean Fluorescence Intensity (MFI)] and *p* values were calculated as initial cut-off (***p < 0.001; two-sided unpaired *t*-test statistical comparisons with Bonferroni corrections).

Since previous human studies (Mori et al., 2016) highlighted the contribution of IL4, IL5 and IL13 in the neural susceptibility to oxidative stress and in the impaired balance between inflammatory and neuroprotective mediators inside the nervous system, a protein array analysis was carried out to better characterize the posterior chamber (vitreous) microenvironment of Reeler mice. The analysis confirmed a selective increase of IL4 [**A**, 2.73 ± 0.01 Mean Fluorescence Intensity (MFI) at p21 (*p* < 0.001) and 2.80 ± 0.04 MFI at p28 (*p* < 0.001)], IL5 [**B**, 2.40 ± 0.02 MFI at p21 (*p* < 0.001) and 2.39 ± 0.02 MFI at p28 (*p* < 0.001)] and IL13 [**C**, 2.43 ± 0.03 MFI p28 (*p* < 0.001)] protein expression, all Reeler *vs*. WT (two-sided unpaired *t*-test statistical comparisons with Bonferroni corrections for number of targets; Figures 9A–C).

## Discussion

The main finding of this study is the immunolocalization of APP/Aβ1-42 and NH_2_htau proteins (AD markers) in retinal tissues from Reelin-deprived mice (Reeler). A possible explanation for this expression of APP/Aβ and tau could be the impaired retinal networking and the onset of the neurodegenerative process due to Reelin absence, most probably associated with an uncontrolled activity of glial and immune cells, as described for the nervous system (Rice et al., 2001). This pathological expression would imply the activation of neurotoxic and/or neuroprotective mechanisms (Rice et al., 2001; Meraz-Rios et al., 2013; Prokop et al., 2013; Micera et al., 2016; Balzamino et al., 2019; Kwon and Koh, 2020). The main localization of APP/Aβ and tau at the GCL suggests the principal involvement of RGCs. Since a slight immunoreactivity was also detected in the entire retina, the activation of additional neuronal, glial and infiltrating immune cells cannot be excluded, although herein not fully investigated. Of interest, the immunoreactivity for these AD markers increased in a time-dependent fashion, starting from p21, implying the development in adulthood. The presence of APP/Aβ1-42 deposits and overexpressed tau in the peripheral retina was previously reported in transgenic AD mice (Ning et al., 2008; Dutescu et al., 2009; Kocherhans et al., 2010; Latina et al., 2021a), and particularly this pathological expression was confirmed in post-mortem AD retinas (den Haan et al., 2018). Gliosis, Drusen and APP/Aβ deposition represent common markers of neurodegeneration in AMD and AD retinas, resulting from damaged RGCs, activated Macroglia (Muller Cells and astrocytes) and reactive Microglia (Lee and Landreth, 2010).

Senile plaques and neurofibrillary tangles have been associated with local/systemic IL1 pathway activation (IL1, IL6, IL8 and IL18) and neuronal death (Strauss et al., 1992; Licastro et al., 2000; Mrak and Griffin, 2005; Eriksson et al., 2011; Sutinen et al., 2012; Salani et al., 2013; Su et al., 2016; Kwon and Koh, 2020). In Reelin retinas, the higher IL1, IL8R and IL18 transcripts’ expression was observed at p28, although starting at p21 (adulthood). This cytokine expression, most probably the result of Reelin deprivation and consequent neurodegeneration, could be the trigger of AD markers’ deposition/expression. The localization of APP/Aβ and tau in these Reelin-deprived retinas might sustain the hypothesis of a link between Reelin and AD markers, as recently reported (Lopez-Font et al., 2022).

As the pathological Aβ deposition and tau overexpression triggers extensive tissue-remodeling, as reported from experimental models and human samples, the potential involvement of few matricellular proteins was investigated in retinal extracts (OPN, CYR61 and MMP9) (Pagenstecher et al., 1998; Bugno et al., 1999; Gu et al., 2005; Scatena et al., 2007; Wung et al., 2007; Capaldo and Nusrat, 2009; Carecchio and Comi, 2011; Muri et al., 2019; Behl et al., 2021; Pentz et al., 2021). A consistent expression of OPN and MMP9 was observed at p28, paralleling the Aβ and NH_2_tau immunoreactivities, in line with previous studies (Agnihotri et al., 2001; Scatena et al., 2007; Chan et al., 2014). Some OPN abilities encompass the proteolytic cleavage of MMP9 that in turn can degrade Aβ, as shown *in vitro* and *in vivo* (Yasuoka et al., 2011; Mori et al., 2016; Morgan et al., 2019). This would imply that OPN and MMP9, in concert with IL6, IL8R and IL18 pathways, might participate in the pathological Aβ deposition and tau expression observed in these Reelin-deprived retinas (Yasuoka et al., 2011; Mori et al., 2016; Balzamino et al., 2019; Morgan et al., 2019). Working as modulators of local neuroinflammation, infiltrating macrophages and reactive microglia might be the main source of MMP9 inside retina; this MMP9 availability might cleave full-length OPN to generate the potent immunoregulatory C-terminal OPN fragments (Scatena et al., 2007; Chan et al., 2014).

Tissue remodeling is often associated with oxidative stress, DNA methylation and activation of several genes involved in cell-to-cell and cell-to-matrix interactions (Morgan et al., 2019; Tamagno et al., 2021). In AD, the oxidative stress has been associated with onset and/or exacerbation of neurodegenerative process. Some explanations for Aβ plaques formation are found in the impairment of ROS/RNS activity and DNA methylation (Morgan et al., 2019; Tamagno et al., 2021). We previously reported that a particular “neuroinflammation” characterizes Reeler retina (Balzamino et al., 2019). While a proinflammatory microenvironment might stimulate APP/Aβ accumulation and tau expression with consequent neuronal degeneration, an excessive neuronal inflammation might also trigger a balancing anti-inflammatory response due to the release of inhibitory molecules aiming to reduce neuronal insult (Kinney et al., 2018; Burgaletto et al., 2020). The low *NFR2*/*KEAP1* ratio observed in these Reelin deprived retinas would confirm a shift of the system toward oxidative stress and neuroprotection (Zhan et al., 2021; Liu et al., 2022). In line with Sha and co-workers, the decrease of *NRF2* might be responsible for the increased *IL6, IL8R and IL18* expressions, as previously described in models of experimental NRF2 deprivation (Saha et al., 2020). The presence of DNA methylation in these Reelin deprived retinas was confirmed by the upregulation of *DNMT3a* transcripts. This result is in line with previous studies on aged brains showing the accumulation of Aβ oligomers upon *IL6* and *DNMT3a* expression (Calvo-Rodríguez et al., 2017; Saha et al., 2020). Not in line with AD observations, the data on *HDAC1*, a zinc-dependent class I histone deacetylase, do not support the presence of an hypermethylated phenotype (Lv et al., 2021).

Morgan and coworkers reported the activation of the innate immune response (mainly complement and TLRs) during the development and progression of neurodegenerative disorders, including AD (Morgan et al., 2019). Herein, the expression of TLR4 in Reeler retina would suggest the direct activation of the innate response to host products generated upon Reelin deprivation (Rice et al., 2001; Balzamino et al., 2019). This effect is not new as DAMP-mediated TLR4 activation was observed in AD tissues characterized by a process of neuronal degeneration associated with DAMPs’ release (Moresco et al., 2011; Gambuzza et al., 2014). Moreover, this TLR4 upregulation might contribute to the cytokine release (IL6), boosting Aβ accumulation at the GCL as observed in brain tissues (Wu et al., 2015; Calvo-Rodríguez et al., 2017). In turn, the overexpression of Aβ aggregates could trigger a long-lasting TLR4 activation with the release of neurotoxic mediators, in addition to the above reported cytokines, promoting neuronal cell death and exacerbating the entire neurodegenerative process (Okun et al., 2011; Trotta et al., 2014; Miron et al., 2018; Hughes et al., 2020).

Up to date, the analysis of vitreous and vitreal reflux represents an valuable alternative to discriminate between vitreoretinal inflammatory and neurodegenerative states, as it was demonstrated that an inflamed retina can be mirrored by vitreal signature (Cacciamani et al., 2016). Our findings on IL4, IL5 and IL13 accumulation in Reeler vitreous can open to multiple aspects in Reelin-deprived scenario. Neuroinflammation occurs when a plethora of inflammatory cytokines (eotaxin-3, granulocyte-macrophage colony-stimulating factor, IL-1β, IL-2, IL-7, IL-10, IL12p70, MIP-1a, TNFβ), including those belonging to the Th2 profile (IL4, IL5, IL13), is released inside the tissues and ocular fluids (Morgan et al., 2019). Although previous studies associated IL4, IL5 and IL13 exclusively with glia and neurons toxicity, others highlighted their ability to stimulate the cleavage of tangles (Morgan et al., 2019). The detection of AD targets (APP/Aβ) was not performed in Reeler vitreous, although it might be of great importance in the contest of human diagnosis, even at early subclinical stages (Meraz-Rios et al., 2013; Prokop et al., 2013; Kwon and Koh, 2020). Neuroinflammation, neurodegeneration and neuroprotection remain important aspects to investigate in human AD progression (Fuster-Matanzo et al., 2013; Cai et al., 2014; Ransohoff, 2016). It remains unclear whether Reelin-deprivation or the consequent inflammation/neurodegeneration might take part in Aβ plaque accumulation in human retina, as observed in the central nervous system (Lopez-Font et al., 2022). Cross-studies of Reelin-deficient and Reelin-overexpressing transgenic mice might sustain AD-genetic mice in understanding how to reduce amyloid plaque formation and counteract the neurodegenerative events occurring in nervous and visual systems (Pujadas et al., 2014).

This model will be useful to provide additional information on the mechanisms inside the retinal network that might occur in AD and AMD neurodegeneration.

## Conclusion

Taken together, Reelin-deficient retinal network could be a useful tool to investigate the age-dependent ocular deterioration (AMD) and better understand the neurodegenerative events of AD. Since the main localization of AD markers was found in RGCs, it is noteworthy to highlight that insulted RGCs and activated Muller Cells are high producers of neurotrophins (NGF and BDNF). This aspect would imply that potential protective routes might be also activated at early stages of Reelin-deprivation, APP/Aβ deposition and tau overexpression (Balzamino et al., 2019; Joshi and Salton, 2022). These findings might provide additional information to sustain the hypothesis that biomarkers of early neurodegeneration can be detected in ocular fluids (AMD) and might be useful for early diagnosis of AD in concert with OCT imaging (Drusen), allowing the possibility to develop alternative therapeutic drugs to counteract neurodegenerative diseases. The participation of Reelin in neurodegenerative disorders is not new, and it is widely accepted that Reelin might overcome neuronal Aβ toxicity, limits APP processing and decreases tau phosphorylation *in situ* (Pujadas et al., 2014; Lussier et al., 2016). The herein proposed Reeler model should not be interpreted as a substitute of AD experimental models, but an “additional tool” to study *in vivo* some aspects of AD neuroinflammation in order to test novel alternative approaches to prevent or slow-down neuroretinal degeneration.

## Data availability statement

The datasets presented in this study can be found in online repositories. The names of the repository/repositories and accession number(s) can be found in the article.

## Ethics statement

This animal study and experimental procedures were reviewed and approved by the Ethical Committee of Tor Vergata University (Rome, Italy) and the Ethical Committee of Campus Bio-Medico University (Rome, Italy), according with ethical standards stated in the Declaration of Helsinki and the ARVO Statement for the Use of Animals in Ophthalmic and Vision Research. All the steps in the procedure were in compliance with the directive 2010/63/EU guidelines, under the authorization provided by the Italian Ministry of Health. All efforts were made to minimize suffering.

## Author contributions

BOB, GE, and RM dissected tissues. BOB and GE performed immunoprecipitation analysis, ELLA analysis, PCR assay, chip-array, and statistical analysis. GA and AM provided tools. BOB and AM conceived the study and supervised all the experiments. BOB, PC, VL, GA, FK, AC, and AM were involved in data analysis. BOB, GA, and AM performed interpretation and wrote the manuscript. All authors read and approved the final manuscript.

## Supplementary material

Supplementary Figure 1 Experiment flow chart. Procedures, sample timing and tissue processing with different platforms.

## References

[B1] Agnihotri R. Crawford H. C. Haro H. Matrisian L. M. Havrda M. C. Liaw L. (2001). Osteopontin, a novel substrate for matrix metalloproteinase-3 (stromelysin-1) and matrix metalloproteinase-7 (matrilysin). *J. Biol. Chem.* 276 28261–28267. 10.1074/jbc.M103608200 11375993

[B2] Amadoro G. Corsetti V. Atlante A. Florenzano F. Capsoni S. Bussani R. (2012). Interaction between NH(2)-tau fragment and Aβ in Alzheimer’s disease mitochondria contributes to the synaptic deterioration. *Neurobiol. Aging* 33 e1–e25. 10.1016/j.neurobiolaging.2011.08.001 21958963

[B3] Amadoro G. Latina V. Balzamino B. O. Squitti R. Varano M. Calissano P. (2021). Nerve growth factor-based therapy in Alzheimer’s disease and age-related macular degeneration. *Front. Neurosci.* 15:735928. 10.3389/fnins.2021.735928 34566573 PMC8459906

[B4] Ashok A. Singh N. Chaudhary S. Bellamkonda V. Kritikos A. E. Wise A. S. (2020). Retinal degeneration and Alzheimer’s disease: An evolving link. *Int. J. Mol. Sci.* 21:7290. 10.3390/ijms21197290 33023198 PMC7582766

[B5] Balzamino B. O. Biamonte F. Esposito G. Marino R. Fanelli F. Keller F. (2014). Characterization of NGF, trkA (NGFR), and p75 (NTR) in retina of mice lacking reelin glycoprotein. *Int. J. Cell Biol.* 2014:725928. 10.1155/2014/725928 24627687 PMC3928862

[B6] Balzamino B. O. Esposito G. Marino R. Keller F. Micera A. (2019). Changes in vitreal protein profile and retina mRNAs in reeler mice: NGF, IL33 and müller cell activation. *PLoS One* 14:e0212732. 10.1371/journal.pone.0212732 30811468 PMC6392297

[B7] Balzamino B. O. Esposito G. Marino R. Keller F. Micera A. (2015). NGF expression in reelin-deprived retinal cells: A potential neuroprotective effect. *Neuromol. Med.* 17 314–325. 10.1007/s12017-015-8360-z 26066836

[B8] Behl T. Kaur G. Sehgal A. Bhardwaj S. Singh S. Buhas C. (2021). Multifaceted role of matrix metalloproteinases in neurodegenerative diseases: Pathophysiological and therapeutic perspectives. *Int. J. Mol. Sci.* 22:1413. 10.3390/ijms22031413 33573368 PMC7866808

[B9] Bugno M. Witek B. Bereta J. Bereta M. Edwards D. R. Kordula T. (1999). Reprogramming of TIMP-1 and TIMP-3 expression profiles in brain microvascular endothelial cells and astrocytes in response to proinflammatory cytokines. *FEBS Lett.* 448 9–14. 10.1016/s0014-5793(99)00323-3 10217399

[B10] Burgaletto C. Munafò A. Di Benedetto G. De Francisci C. Caraci F. Di Mauro R. (2020). The immune system on the TRAIL of Alzheimer’s disease. *J. Neuroinflammation* 17:298. 10.1186/s12974-020-01968-1 33050925 PMC7556967

[B11] Cacciamani A. Parravano M. Scarinci F. Esposito G. Varano M. Micera A. (2016). A simple spontaneous vitreal reflux collecting procedure during intravitreal injection: Set-up and validation studies. *Curr. Eye Res.* 41 971–976. 10.3109/02713683.2015.1080282 26470652

[B12] Cai Z. Hussain M. D. Yan L. J. (2014). Microglia, neuroinflammation, and beta-amyloid protein in Alzheimer’s disease. *Int. J. Neurosci.* 124 307–321. 10.3109/00207454.2013.833510 23930978

[B13] Calsolaro V. Edison P. (2016). Neuroinflammation in Alzheimer’s disease: Current evidence and future directions. *Alzheimers Dement.* 12 719–732. 10.1016/j.jalz.2016.02.010 27179961

[B14] Calvo-Rodríguez M. de la Fuente C. García-Durillo M. García-Rodríguez C. Villalobos C. Núñez L. (2017). Aging and amyloid β oligomers enhance TLR4 expression, LPS-induced Ca2+ responses, and neuron cell death in cultured rat hippocampal neurons. *J. Neuroinflammation* 14:24. 10.1186/s12974-017-0802-0 28143556 PMC5282876

[B15] Cameron B. Landreth G. E. (2010). Inflammation, microglia, and Alzheimer’s disease. *Neurobiol. Dis.* 37 503–509. 10.1016/j.nbd.2009.10.006 19833208 PMC2823849

[B16] Capaldo C. T. Nusrat A. (2009). Cytokine regulation of tight junctions. *Biochim. Biophys. Acta* 1788 864–5871. 10.1016/j.bbamem.2008.08.027 18952050 PMC2699410

[B17] Carecchio M. Comi C. (2011). The role of osteopontin in neurodegenerative diseases. *J. Alzheimers Dis.* 25 179–185. 10.3233/JAD-2011-102151 21358042

[B18] Chan J. L. Reeves T. M. Phillips L. L. (2014). Osteopontin expression in acute immune response mediates hippocampal synaptogenesis and adaptive outcome following cortical brain injury. *Exp. Neurol.* 261 757–771. 10.1016/j.expneurol.2014.08.015 25151457 PMC4262258

[B19] Chu E. M. Kolappan M. Barnes T. R. Joyce E. M. Ron M. A. (2012). A window into the brain: An in vivo study of the retina in schizophrenia using optical coherence tomography. *Psychiatry Res.* 203 89–94. 10.1016/j.pscychresns.2011.08.011 22917503 PMC4024658

[B20] Cordeiro M. F. Guo L. Coxon K. M. Duggan J. Nizari S. Normando E. M. (2010). Imaging multiple phases of neurodegeneration: A novel approach to assessing cell death in vivo. *Cell Death Dis.* 1:e3. 10.1038/cddis.2009.3 21364622 PMC3032512

[B21] Corsetti V. Borreca A. Latina V. Giacovazzo G. Pignataro A. Krashia P. (2020). Passive immunotherapy for N-truncated NH2htau ameliorates the cognitive deficits in two mouse Alzheimer’s disease models. *Brain Commun.* 2:fcaa039. 10.1093/braincomms/fcaa039 32954296 PMC7425324

[B22] Cuchillo-Ibáñez I. Balmaceda V. Botella-López A. Rabano A. Avila J. Sáez-Valero J. (2013). Beta-amyloid impairs reelin signaling. *PLoS One* 8:e72297. 10.1371/journal.pone.0072297 23951306 PMC3741172

[B23] Czakó C. Kovács T. Ungvari Z. Csiszar A. Yabluchanskiy A. Conley S. (2020). Retinal biomarkers for Alzheimer’s disease and vascular cognitive impairment and dementia (VCID): Implication for early diagnosis and prognosis. *Geroscience* 42 1499–1525. 10.1007/s11357-020-00252-7 33011937 PMC7732888

[B24] den Haan J. Morrema T. Verbraak F. D. de Boer J. F. Scheltens P. Rozemuller A. J. (2018). Amyloid-beta and phosphorylated NH2htau in post-mortem Alzheimer’s disease retinas. *Acta Neuropathol. Commun.* 6:147. 10.1186/s40478-018-0650-x 30593285 PMC6309096

[B25] Durakoglugil M. S. Chen Y. White C. L. Kavalali E. T. Herz J. (2009). “Reelin signaling antagonizes beta-amyloid at the synapse,” in *Proceedings of the national academy of sciences of the United States of America*, Washington, DC. 10.1073/pnas.0908176106 PMC2747222 19805234

[B26] Dutescu R. M. Li Q. X. Crowston J. Masters C. L. Baird P. N. Culvenor J. G. (2009). Amyloid precursor protein processing and retinal pathology in mouse models of Alzheimer’s disease. *Graefes Arch. Clin. Exp. Ophthalmol.* 247 1213–1221. 10.1007/s00417-009-1060-3 19271231

[B27] Eriksson P. S. Perfilieva E. Björk-Eriksson T. Alborn A. M. Nordborg C. Peterson D. A. (1998). Neurogenesis in the adult human hippocampus. *Nat. Med.* 4 1313–1317. 9809557 10.1038/3305

[B28] Eriksson U. K. Pedersen N. L. Reynolds C. A. Hong M. G. Prince J. A. Gatz M. (2011). Associations of gene sequence variation and serum levels of C-reactive protein and interleukin-6 with Alzheimer’s disease and dementia. *J. Alzheimers Dis.* 23 361–369. 10.3233/JAD-2010-101671 21116047 PMC3237048

[B29] Fuster-Matanzo A. Llorens-Martín M. Hernández F. Avila J. (2013). Role of neuroinflammation in adult neurogenesis and Alzheimer disease: Therapeutic approaches. *Mediators Inflamm.* 2013:260925. 10.1155/2013/260925 23690659 PMC3649701

[B30] Galasko D. R. Shaw L. M. (2017). Alzheimer disease: CSF biomarkers for Alzheimer disease – approaching consensus. *Nat. Rev. Neurol.* 13 131–132. 10.1038/nrneurol.2017.11 28155892 PMC5912691

[B31] Gambuzza M. E. Sofo V. Salmeri F. M. Soraci L. Marino S. Bramanti P. (2014). Toll-like receptors in Alzheimer’s disease: A therapeutic perspective. *CNS Neurol. Disord. Drug Targets* 13 1542–1558. 10.2174/1871527313666140806124850 25106635

[B32] Gu Z. Cui J. Brown S. Fridman R. Mobashery S. Strongin A. Y. (2005). A highly specific inhibitor of matrix metalloproteinase-9 rescues laminin from proteolysis and neurons from apoptosis in transient focal cerebral ischemia. *J. Neurosci.* 25 6401–6408. 10.1523/JNEUROSCI.1563-05.2005 16000631 PMC6725288

[B33] Gupta V. B. Chitranshi N. den Haan J. Mirzaei M. You Y. Lim J. K. (2021). Retinal changes in Alzheimer’s disease- integrated prospects of imaging, functional and molecular advances. *Prog. Retin. Eye Res.* 82:100899. 10.1016/j.preteyeres.2020.100899 32890742

[B34] Hirota Y. Kubo K. Katayama K. Honda T. Fujino T. Yamamoto T. T. (2015). Reelin receptors ApoER2 and VLDLR are expressed in distinct spatiotemporal patterns in developing mouse cerebral cortex. *J. Comp. Neurol.* 523:4. 10.1002/cne.23691 25308109

[B35] Hoe H. S. Lee K. J. Carney R. S. Lee J. Markova A. Lee J. Y. (2009). Interaction of reelin with amyloid precursor protein promotes neurite outgrowth. *J. Neurosci.* 29 7459–7473. 10.1523/JNEUROSCI.4872-08.2009 19515914 PMC2759694

[B36] Hughes C. Choi M. L. Yi J. H. Kim S. C. Drews A. George-Hyslop P. S. (2020). Beta amyloid aggregates induce sensitised TLR4 signalling causing long-term potentiation deficit and rat neuronal cell death. *Commun. Biol.* 3:79. 10.1038/s42003-020-0792-9 32071389 PMC7028984

[B37] Jack C. R. Jr. Wiste H. J. Weigand S. D. Therneau T. M. Knopman D. S. Lowe V. (2017). Age-specific and sex-specific prevalence of cerebral β-amyloidosis, NH2htauopathy, and neurodegeneration in cognitively unimpaired individuals aged 50-95 years: A cross-sectional study. *Lancet Neurol.* 16 435–444. 10.1016/S1474-4422(17)30077-7 28456479 PMC5516534

[B38] Javaid F. Z. Brenton J. Guo L. Cordeiro M. F. (2016). Visual and ocular manifestations of Alzheimer’s disease and their use as biomarkers for diagnosis and progression. *Front. Neurol.* 7:55. 10.3389/fneur.2016.00055 27148157 PMC4836138

[B39] Jensen P. Patel B. Smith S. Sabnis R. Kaboord B. (2021). Improved immunoprecipitation to mass spectrometry method for the enrichment of low-abundant protein targets. *Methods Mol. Biol.* 2261 229–246. 10.1007/978-1-0716-1186-9_14 33420993

[B40] Joshi R. Salton S. (2022). Neurotrophin crosstalk in the etiology and treatment of neuropsychiatric and neurodegenerative disease. *Front. Mol. Neurosci.* 15:932497. 10.3389/fnmol.2022.932497 35909451 PMC9335126

[B41] Kinney J. W. Bemiller S. M. Murtishaw A. S. Leisgang A. M. Salazar A. M. Lamb B. T. (2018). Inflammation as a central mechanism in Alzheimer’s disease. *Alzheimers Dement.* 4 575–590. 10.1016/j.trci.2018.06.014 30406177 PMC6214864

[B42] Kocherhans S. Madhusudan A. Doehner J. Breu K. S. Nitsch R. M. Fritschy J. M. (2010). Reduced Reelin expression accelerates amyloid-beta plaque formation and NH2htau pathology in transgenic Alzheimer’s disease mice. *J. Neurosci.* 30 9228–9240. 10.1523/JNEUROSCI.0418-10.2010 20610758 PMC6632461

[B43] Koronyo-Hamaoui M. Koronyo Y. Ljubimov A. V. Miller C. A. Ko M. K. Black K. L. (2011). Identification of amyloid plaques in retinas from Alzheimer’s patients and noninvasive in vivo optical imaging of retinal plaques in a mouse model. *Neuroimage* 54(Suppl. 1) S204–S217. 10.1016/j.neuroimage.2010.06.020 20550967 PMC2991559

[B44] Krstic D. Pfister S. Notter T. Knuesel I. (2013). Decisive role of Reelin signaling during early stages of Alzheimer’s disease. *Neuroscience* 246 108–116. 10.1016/j.neuroscience.2013.04.042 23632168

[B45] Kwon H. S. Koh S. H. (2020). Neuroinflammation in neurodegenerative disorders: The roles of microglia and astrocytes. *Transl. Neurodegener.* 9:42. 10.1186/s40035-020-00221-2 33239064 PMC7689983

[B46] Latina V. Giacovazzo G. Calissano P. Atlante A. La Regina F. Malerba F. (2021a). Tau cleavage contributes to cognitive dysfunction in strepto-zotocin-induced sporadic Alzheimer’s disease (sAD) mouse model. *Int. J. Mol. Sci.* 22:12158. 10.3390/ijms222212158 34830036 PMC8618605

[B47] Latina V. Giacovazzo G. Cordella F. Balzamino B. O. Micera A. Varano M. (2021b). Systemic delivery of a specific antibody targeting the pathological N-terminal truncated NH2htau peptide reduces retinal degeneration in a mouse model of Alzheimer’s disease. *Acta Neuropathol. Commun.* 9:38. 10.1186/s40478-021-01138-1 33750467 PMC7942014

[B48] Lee C. Y. Landreth G. E. (2010). The role of microglia in amyloid clearance from the AD brain. *J. Neural Transm.* 117 949–960. 10.1007/s00702-010-0433-4 20552234 PMC3653296

[B49] Licastro F. Pedrini S. Caputo L. Annoni G. Davis L. J. Ferri C. (2000). Increased plasma levels of interleukin-1, interleukin-6 and alpha-1-antichymotrypsin in patients with Alzheimer’s disease: Peripheral inflammation or signals from the brain? *J. Neuroimmunol.* 103 97–102. 10.1016/s0165-5728(99)00226-x 10674995

[B50] Liu S. Pi J. Zhang Q. (2022). Signal amplification in the KEAP1-NRF2-ARE antioxidant response pathway. *Redox Biol.* 54:102389. 10.1016/j.redox.2022.102389 35792437 PMC9287733

[B51] Lopez-Font I. Lennol M. P. Iborra-Lazaro G. Zetterberg H. Blennow K. Sáez-Valero J. (2022). Altered balance of reelin proteolytic fragments in the cerebrospinal fluid of Alzheimer’s disease patients. *Int. J. Mol. Sci.* 23:7522. 10.3390/ijms23147522 35886870 PMC9318932

[B52] Lugert S. Basak O. Knuckles P. Haussler U. Fabel K. Götz M. (2010). Quiescent and active hippocampal neural stem cells with distinct morphologies respond selectively to physiological and pathological stimuli and aging. *Cell Stem Cell* 6 445–456. 10.1016/j.stem.2010.03.017 20452319

[B53] Lussier A. L. Weeber E. J. Rebeck G. W. (2016). Reelin proteolysis affects signaling related to normal synapse function and neurodegeneration. *Front. Cell. Neurosci.* 10:75. 10.3389/fncel.2016.00075 27065802 PMC4809875

[B54] Lv L. Zhang D. Hua P. Yang S. (2021). The glial-specific hypermethylated 3’ untranslated region of histone deacetylase 1 may modulates several signal pathways in Alzheimer’s disease. *Life Sci.* 265:118760. 10.1016/j.lfs.2020.118760 33212149

[B55] Mei X. Yang M. Zhu L. Zhou Q. Li X. Chen Z. (2020). Retinal levels of amyloid beta correlate with cerebral levels of amyloid beta in young APPswe/PS1dE9 transgenic mice before onset of Alzheimer’s disease. *Behav. Neurol.* 2020:1574816. 10.1155/2020/1574816 33029254 PMC7532376

[B56] Meraz-Rios M. A. Toral-Rios D. Franco-Bocanegra D. Villeda-Hernández J. Campos-Peña V. (2013). Inflammatory process in Alzheimer’s disease. *Front. Integr. Neurosci.* 7:59. 10.3389/fnint.2013.00059 23964211 PMC3741576

[B57] Micera A. Balzamino B. O. Biamonte F. Esposito G. Marino R. Fanelli F. (2016). Current progress of Reelin in development, inflammation and tissue remodeling: From nervous to visual systems. *Curr. Mol. Med.* 16 620–630. 27494703

[B58] Micera A. Bruno L. Cacciamani A. Rongioletti M. Squitti R. (2019). Alzheimer’s disease and retinal degeneration: A glimpse at essential trace metals in ocular fluids and tissues. *Curr. Alzheimer Res.* 16 1073–1083. 10.2174/1567205016666191023114015 31642780

[B59] Miron J. Picard C. Frappier J. Dea D. Théroux L. Poirier J. (2018). TLR4 gene expression and pro-inflammatory cytokines in Alzheimer’s disease and in response to hippocampal deafferentation in rodents. *J. Alzheimers Dis.* 63 1547–1556. 10.3233/JAD-171160 29782315

[B60] Mirzaei N. Shi H. Oviatt M. Doustar J. Rentsendorj A. Fuchs D. T. (2020). Alzheimer’s retinopathy: Seeing disease in the eyes. *Front. Neurosci.* 14:921. 10.3389/fnins.2020.00921 33041751 PMC7523471

[B61] Moresco E. M. LaVine D. Beutler B. (2011). Toll-like receptors. *Curr. Biol.* 21 R488–R493. 10.1016/j.cub.2011.05.039 21741580

[B62] Morgan A. R. Touchard S. Leckey C. O’Hagan C. Nevado-Holgado A. J. Nima Consortium (2019). Inflammatory biomarkers in Alzheimer’s disease plasma. *Alzheimers Dement.* 15 776–787. 10.1016/j.jalz.2019.03.007 31047856 PMC6565806

[B63] Mori S. Maher P. Conti B. (2016). Neuroimmunology of the interleukins 13 and 4. *Brain Sci.* 6:18. 10.3390/brainsci6020018 27304970 PMC4931495

[B64] Mrak R. E. Griffin W. S. (2005). Potential inflammatory biomarkers in Alzheimer’s disease. *J. Alzheimers Dis.* 8 369–375. 10.3233/jad-2005-8406 16556968

[B65] Muri L. Leppert D. Grandgirard D. Leib S. L. (2019). MMPs and ADAMs in neurological infectious diseases and multiple sclerosis. *Cell. Mol. Life Sci.* 76 3097–3116. 10.1007/s00018-019-03174-6 31172218 PMC7079810

[B66] Ning A. Cui J. To E. Ashe K. H. Matsubara J. (2008). Amyloid-beta deposits lead to retinal degeneration in a mouse model of Alzheimer disease. *Invest. Ophthalmol. Vis. Sci.* 49 5136–5143. 10.1167/iovs.08-1849 18566467 PMC3947384

[B67] Niu S. Yabut O. D’Arcangelo G. (2008). The reelin signaling pathway promotes dendritic spine development in hippocampal neurons. *J. Neurosci.* 28 10339–10348. 10.1523/JNEUROSCI.1917-08.2008 18842893 PMC2572775

[B68] Notter T. Knuesel I. (2013). Reelin immunoreactivity in neuritic varicosities in the human hippocampal formation of non-demented subjects and Alzheimer’s disease patients. *Acta Neuropathol. Commun.* 1:27. 10.1186/2051-5960-1-27 24252415 PMC3893416

[B69] Oddo S. Caccamo A. Shepherd J. D. Murphy M. P. Golde T. E. Kayed R. (2003). Triple-transgenic model of Alzheimer’s disease with plaques and tangles: Intracellular Abeta and synaptic dysfunction. *Neuron* 39 409–421. 10.1016/s0896-6273(03)00434-3 12895417

[B70] Okun E. Griffioen K. J. Mattson M. P. (2011). Toll-like receptor signaling in neural plasticity and disease. *Trends Neurosci.* 34 269–281. 10.1016/j.tins.2011.02.005 21419501 PMC3095763

[B71] Pagenstecher A. Stalder A. K. Kincaid C. L. Shapiro S. D. Campbell I. L. (1998). Differential expression of matrix metalloproteinase and tissue inhibitor of matrix metalloproteinase genes in the mouse central nervous system in normal and inflammatory states. *Am. J. Pathol.* 152 729–741. 9502415 PMC1858390

[B72] Pentz R. Iulita M. F. Ducatenzeiler A. Bennett D. A. Cuello A. C. (2021). The human brain NGF metabolic pathway is impaired in the pre-clinical and clinical continuum of Alzheimers disease. *Mol. Psychiatry* 26 6023–6037. 10.1038/s41380-020-0797-2 32488129 PMC10194044

[B73] Pfaffl M. W. (2001). A new mathematical model for relative quantification in real-time RT-PCR. *Nucleic Acids Res.* 29:e45. 10.1093/nar/29.9.e45 11328886 PMC55695

[B74] Platania C. Fisichella V. Fidilio A. Geraci F. Lazzara F. Leggio G. M. (2017). Topical ocular delivery of TGF-β1 to the back of the eye: Implications in age-related neurodegenerative diseases. *Int. J. Mol. Sci.* 18:2076. 10.3390/ijms18102076 28973964 PMC5666758

[B75] Prokop S. Miller K. R. Heppner F. L. (2013). Microglia actions in Alzheimer’s disease. *Acta Neuropathol.* 126 461–477. 10.1007/s00401-013-1182-x 24224195

[B76] Pujadas L. Rossi D. Andrés R. Teixeira C. M. Serra-Vidal B. Parcerisas A. (2014). Reelin delays amyloid-beta fibril formation and rescues cognitive deficits in a model of Alzheimer’s disease. *Nat. Commun.* 5:3443. 10.1038/ncomms4443 24599114

[B77] Ransohoff R. M. (2016). How neuroinflammation contributes to neurodegeneration. *Science* 353 777–783. 10.1126/science.aag2590 27540165

[B78] Rice D. S. Nusinowitz S. Azimi A. M. Martínez A. Soriano E. Curran T. (2001). The reelin pathway modulates the structure and function of retinal synaptic circuitry. *Neuron* 31 929–941. 10.1016/s0896-6273(01)00436-6 11580894

[B79] Rice H. C. Young-Pearse T. L. Selkoe D. J. (2013). Systematic evaluation of candidate ligands regulating ectodomain shedding of amyloid precursor protein. *Biochemistry* 52 3264–3277. 10.1021/bi400165f 23597280 PMC3809327

[B80] Saha S. Buttari B. Panieri E. Profumo E. Saso L. (2020). An overview of nrf2 signaling pathway and its role in inflammation. *Molecules* 25:5474. 10.3390/molecules25225474 33238435 PMC7700122

[B81] Salani F. Ciaramella A. Bizzoni F. Assogna F. Caltagirone C. Spalletta G. (2013). Increased expression of interleukin-18 receptor in blood cells of subjects with mild cognitive impairment and Alzheimer’s disease. *Cytokine* 61 360–363. 10.1016/j.cyto.2012.11.001 23201485

[B82] Scatena M. Liaw L. Giachelli C. M. (2007). Osteopontin: A multifunctional molecule regulating chronic inflammation and vascular disease. *Arterioscler. Thromb. Vasc. Biol.* 27 2302–2309. 10.1161/ATVBAHA.107.144824 17717292

[B83] Schultz N. Byman E. Bank Wennström M. (2020). Levels of retinal amyloid-β correlate with levels of retinal IAPP and hippocampal amyloid-β in neuropathologically evaluated individuals. *J. Alzheimers Dis.* 73 1201–1209. 10.3233/JAD-190868 31884473 PMC7081096

[B84] Strauss S. Bauer J. Ganter U. Jonas U. Berger M. Volk B. (1992). Detection of interleukin-6 and alpha 2-macroglobulin immunoreactivity in cortex and hippocampus of Alzheimer’s disease patients. *Lab. Invest.* 66 223–230. 1370967

[B85] Su F. Bai F. Zhang Z. (2016). Inflammatory cytokines and Alzheimer’s disease: A review from the perspective of genetic polymorphisms. *Neurosci. Bull.* 32 469–480. 10.1007/s12264-016-0055-4 27568024 PMC5563762

[B86] Sutinen E. M. Pirttilä T. Anderson G. Salminen A. Ojala J. O. (2012). Pro-inflammatory interleukin-18 increases Alzheimer’s disease-associated amyloid-β production in human neuron-like cells. *J. Neuroinflammation* 9:199. 10.1186/1742-2094-9-199 22898493 PMC3458954

[B87] Tamagno E. Guglielmotto M. Vasciaveo V. Tabaton M. (2021). Oxidative stress and beta amyloid in Alzheimer’s disease. Which comes first: The chicken or the egg? *Antioxidants* 10:1479. 10.3390/antiox10091479 34573112 PMC8468973

[B88] Trotta T. Porro C. Calvello R. Panaro M. A. (2014). Biological role of Toll-like receptor-4 in the brain. *J. Neuroimmunol.* 268 1–12. 10.1016/j.jneuroim.2014.01.014 24529856

[B89] Vélez-Bermúdez I. C. Salazar-Henao J. E. Riera M. Caparros-Ruiz D. Schmidt W. (2022). Protein and antibody purification followed by immunoprecipitation of MYB and GATA zinc finger-type maize proteins with magnetic beads. *STAR Protoc.* 3:101449. 10.1016/j.xpro.2022.101449 35693212 PMC9184801

[B90] Wu D. Xie J. Wang X. Zou B. Yu Y. Jing T. (2015). Micro-concentration lipopolysaccharide as a novel stimulator of megakaryocytopoiesis that synergizes with IL-6 for platelet production. *Sci. Rep.* 5:13748. 10.1038/srep13748 26330186 PMC4557119

[B91] Wung J. K. Perry G. Kowalski A. Harris P. L. Bishop G. M. Trivedi M. A. (2007). Increased expression of the remodeling- and tumorigenic-associated factor osteopontin in pyramidal neurons of the Alzheimer’s disease brain. *Curr. Alzheimer Res.* 4 67–72. 10.2174/156720507779939869 17316167

[B92] Yasuoka S. Kawanokuchi J. Parajuli B. Jin S. Doi Y. Noda M. (2011). Production and functions of IL-33 in the central nervous system. *Brain Res.* 1385 8–17. 10.1016/j.brainres.2011.02.045 21349253

[B93] Yu N. N. Tan M. S. Yu J. T. Xie A. M. Tan L. (2016). The role of reelin signaling in Alzheimer’s disease. *Mol. Neurobiol.* 53 5692–5700. 10.1007/s12035-015-9459-9 26491027

[B94] Zhan X. Li J. Zhou T. (2021). Targeting Nrf2-mediated oxidative stress response signaling pathways as new therapeutic strategy for pituitary adenomas. *Front. Pharmacol.* 12:565748. 10.3389/fphar.2021.565748 33841137 PMC8024532

