# Supplementary figures and images for: Morphological and biomolecular targets in retina and vitreous from Reelin-deficient mice (Reeler): Potential implications for age-related macular degeneration in Alzheimer’s dementia
Source: Front Aging Neurosci. 2022 Nov 16;14:1015359. doi: 10.3389/fnagi.2022.1015359 (PMC9708893; doi:10.3389/fnagi.2022.1015359)

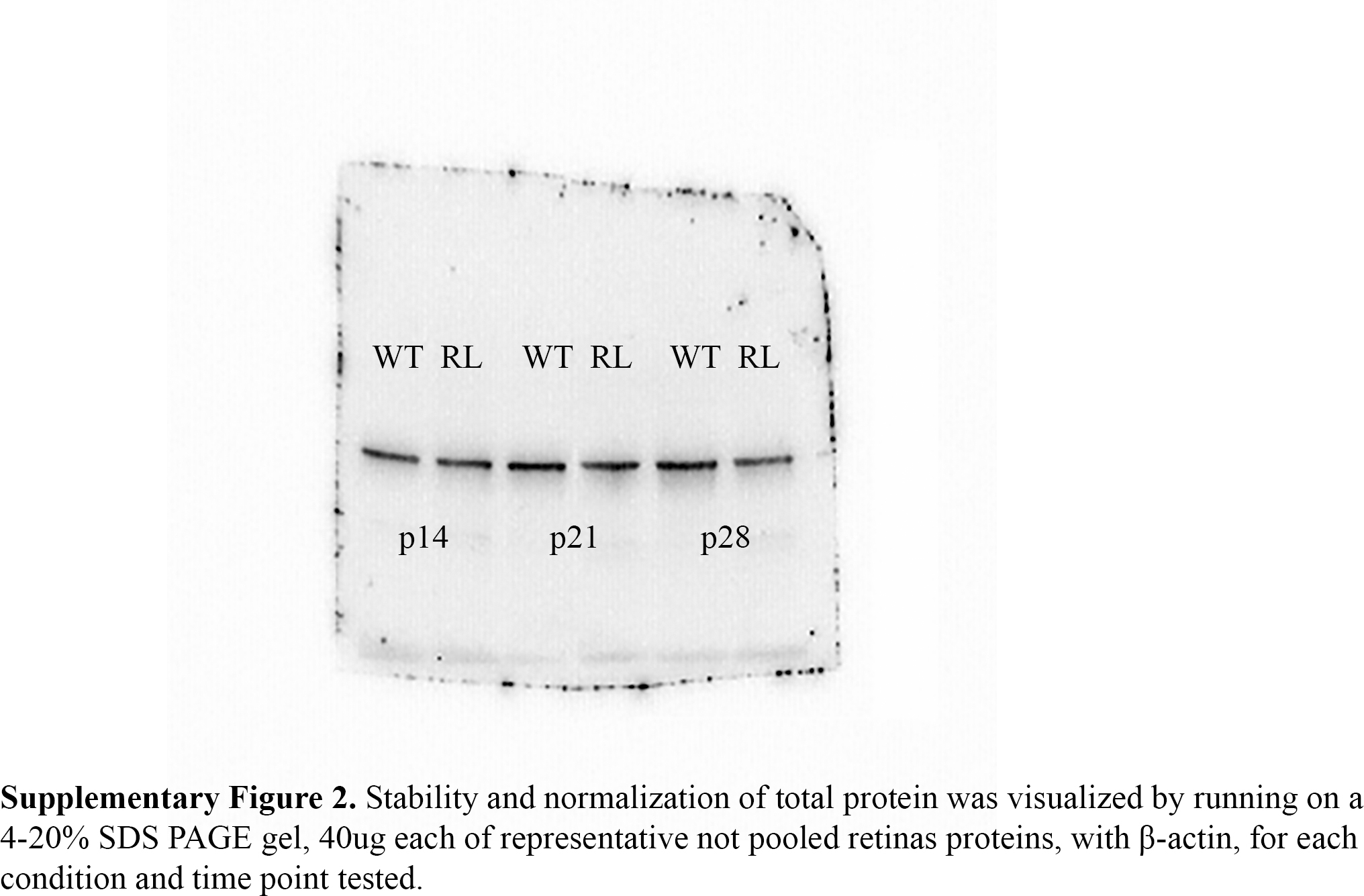

Supplement: Supplementary file 1 [file Image_2.JPEG]

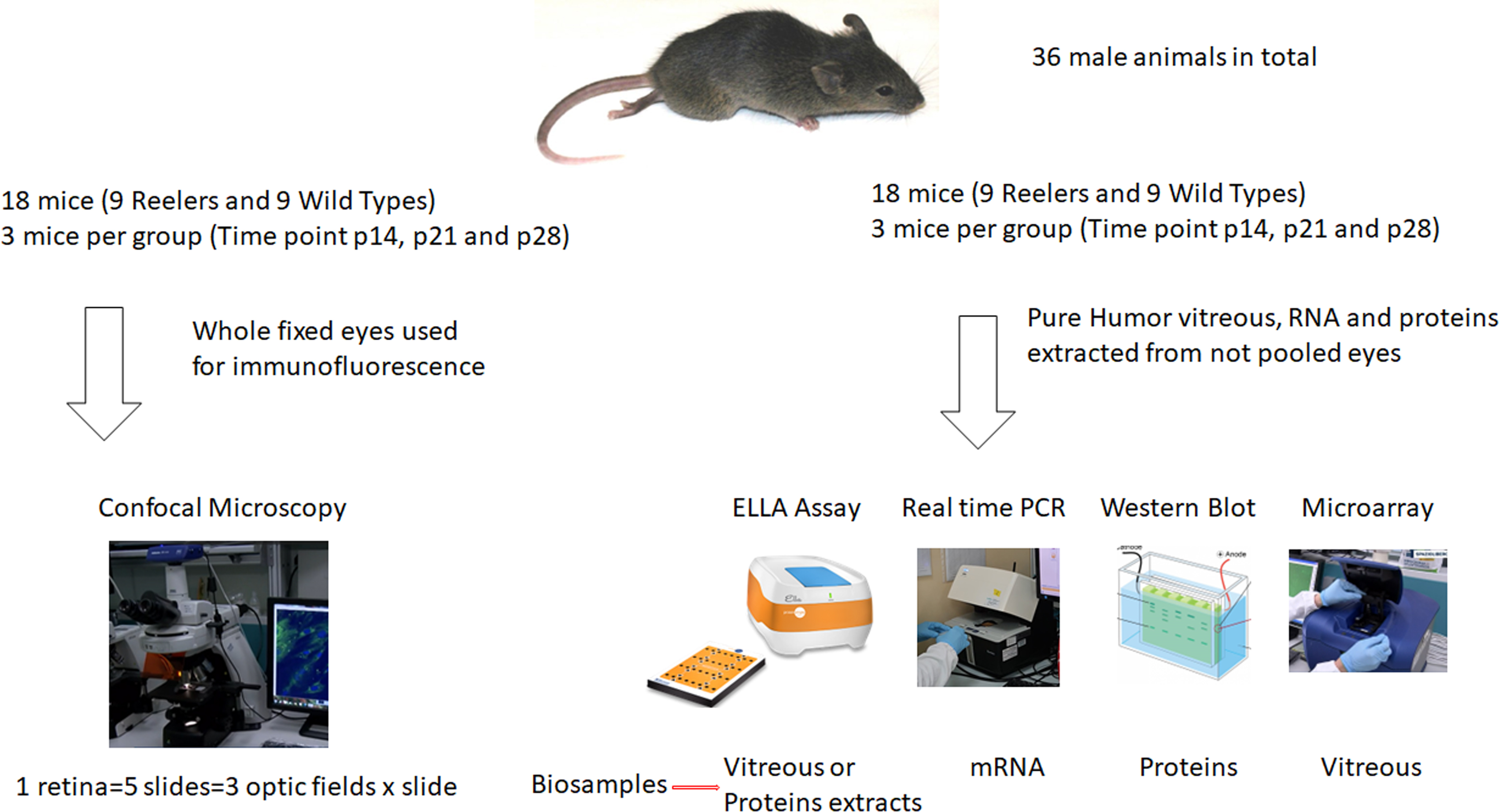

Supplement: Supplementary Figure 1 — Experiment flow chart. Procedures, sample timing and tissue processing with different platforms. [file Image_1.JPEG]
